# The association of genetic variants in Krüppel-like factor 11 and Type 2 diabetes in the Japanese population

**DOI:** 10.1111/j.1464-5491.2007.02315.x

**Published:** 2008-01

**Authors:** T Tanahashi, K Shinohara, P Keshavarz, Y Yamaguchi, K Miyawaki, K Kunika, M Moritani, N Nakamura, T Yoshikawa, H Shiota, H Inoue, M Itakura

**Affiliations:** Division of Genetic Information, Institute for Genome Research, University of Tokushima Tokushima; * Department of Endocrinology and Metabolism, Kyoto Prefectural University of Medicine Graduate School of Medical Sciences Kyoto; † Department of Ophthalmology and Visual Neuroscience, Institute for Health Biosciences, University of Tokushima Tokushima, Japan

**Keywords:** association study, Japanese, *KLF11*, Type 2 diabetes

## Abstract

**Aims:**

Krüppel-like factor 11 (*KLF11*) is a transcriptional factor of the zinc finger domain family that regulates the expression of insulin. In North European populations, its common functional variant Q62R (rs35927125) is a strong genetic factor for Type 2 diabetes (*P* = 0.00033, odds ratio for G allele = 1.29, 95% CI 1.12–1.49). We examined the contribution of *KLF11* variants to the susceptibility to Type 2 diabetes in a Japanese population.

**Methods:**

By re-sequencing Japanese individuals (*n* = 24, partly 96), we screened all four exons, exon/intron boundaries and flanking regions of *KLF11*. Verified single nucleotide polymorphisms (SNPs) were genotyped in 731 initial samples (369 control and 362 case subjects). Subsequently, we tested for association in 1087 samples (524 control and 563 case subjects), which were collected in different districts of Japan from the initial samples.

**Results:**

We identified eight variants, including a novel A/C variant on intron 3, but no mis-sense mutations. In an association study, we failed to find any significant result of SNPs (minor allele frequency 8.2–46.2%) after correcting for multiple testing. Similarly, no haplotypes were associated with Type 2 diabetes. It is notable that the G allele in rs35927125 was completely absent in 1818 Japanese individuals.

**Conclusions:**

Genetic variants in *KLF11* are unlikely to have a major effect of Type 2 diabetes in the Japanese population, although they were significantly associated in North European populations. These observations might help to determine the role of *KLF11* variants in Type 2 diabetes in different populations.

## Introduction

The Krüppel-like factor (KLF) family is characterized by the presence of three highly homologous Cys2/His2 type zinc finger domains near the C-terminal region. It is a transcription factor involved in cellular development and differentiation. To date, 17 members of the KLF family have been identified, and each could play an important role in mammalian cells [1, 2]. *KLF2* is a negative regulator of adipocyte differentiation [3], and *KLF5* regulates the differentiation of smooth muscle and adipose tissue [4].

Recently, it has been shown that *KLF11*, inducible by transforming growth factor (TGF)-β, regulates exocrine cell growth and behaves as a tumour suppressor in pancreatic cancer [5]. Molecular mechanistic studies in pancreatic B-cells showed that the *KLF11* gene is a glucose-induced regulator of the insulin gene. Various homozygous and heterozygous variants, including deletion, insertion and mis-sense mutations, have been detected within this gene, and its common functional variant Q62R (rs35927125) is significantly associated with Type 2 diabetes in North European populations [6]. This raises the possibility that *KLF11* is one of the key genes for predisposition to diabetes that affects insulin secretion.

The disease susceptibility variants modestly affect the risk of Type 2 diabetes, and a large study population is required to ensure substantial significance. So far, robust reproducible associations have been documented in a few genes, such as the P12A polymorphism in peroxisome proliferator-activated receptor γ (encoded by *PPARγ*) [7], the E23K polymorphism in *KCNJ11*, which encodes the ATP-sensitive K^+^ channel Kir6.2 [8], and common variants in the transcription factor 7-like 2 (*TCF7L2*) gene [9].

In contrast, some association studies have given conflicting results, with initial findings not confirmed in all subsequent studies. More recently, a study of 8676 Caucasian individuals failed to replicate the association between *KLF11* variants and Type 2 diabetes [10], despite an association in North European populations [6]. To analyse the substantial effect of genetic variants, it is necessary to have independent reproducible analysis in multiple ethnic groups.

In the light of the potential role of *KLF11* in insulin action, we present a detailed re-examination of the *KLF11* variants and test the possible association with Type 2 diabetes in 1818 Japanese participants.

## Patients and methods

The World Health Organization criteria were used for the classification of Type 2 diabetic patients and healthy control subjects [11]. Type 2 diabetes was clinically defined as a gradual onset of the disease in adulthood, requiring medication. Patients with mitochondrial diseases or maturity-onset diabetes of the young (MODY) were excluded. Using birthplace information, all subjects were of full Japanese ancestry. A description of cases and control subjects is provided in Table 1.

**Table 1:** Clinical characteristics of 1818 Japanese subjects in the association study (initial, replication and whole samples)

		Initial samples (*n* = 731)		Replication samples (*n* = 1087)		Whole samples (*n* = 1818)	
				
Parameters		Cases (Tokushima)	Control subjects (Tokushima)	Cases (Kansai)	Control subjects	Cases	Control subjects
*n*	(male/female)	362 (183/179)	369 (116/253)	563 (285/278)	524* (320/204)	925 (468/457)	893 (436/457)
Age	(years)	62.1 ± 10.0	36.4 ± 1216	64.1 ± 10.0	39.1 ± 12.8	63.4 ± 10.1	38.0 ± 12.6
BMI	(kg/m^2^)	23.6 ± 3.23	22.0 ± 3.1	23.4 ± 3.4	22.2 ± 2.8	23.4 ± 3.4	22.1 ± 3.0
HbA_1c_	(%)	7.1 ± 1.32	4.7 ± 0.33	7.4 ± 1.35	4.9 ± 0.32	7.4 ± 1.36	4.8 ± 0.33
Age at onset	< 40 years (%)	128 (35.4)	—	82 (14.6)	—	210 (22.7)	—
	> 40 years (%)	232 (64.1)	—	479 (85.1)	—	711 (76.9)	—
	Unknown (%)	2 (0.5)	—	2 (0.3)	—	4 (0.4)	—
Positive family history	(First-degree relatives, %)	155 (42.8)	—	201 (35.7)	—	356 (38.5)	—

* 507 control samples (96.7%) were recruited through PSC.

Age, BMI and HbA_1c_ are represented as the means ± sd.

BMI, body mass index; HbA_1c_, glycated haemoglobin; PSC, Pharma SNP consortium; sd, standard deviation.

We prepared two independent samples of 1818 Japanese individuals (925 cases and 893 control subjects). In 731 initial samples, 362 Type 2 diabetic patients and 369 healthy control subjects were recruited from the outpatient clinic of Tokushima University Hospital. They are all residents of Shikoku Island, one of the four main islands of Japan.

Even within relatively homogenous genetic isolates, such as Icelanders, there is a substantial divergence in allele frequencies among geographical areas [12]. To prevent the substantial divergence in allele frequencies from population stratification, we replicated the association in the other unrelated 1087 samples of 563 cases and 524 control subjects. In the replication test, cases were collected from Kyoto Prefectural University Hospital and its related hospitals. They are all residents of the Kansai district located in the western part of the main island of Japan, 130 miles away from Shikoku Island, by sea. Non-diabetic control subjects were recruited from the general population. A large number of control subjects (96.7%) was recruited through the Pharma SNP consortium (PSC) in Japan (http://www.jpma.or.jp/psc/frame-e.html). Through the consortium, their health and absence of diabetes were intensively checked.

The study protocol was approved by the Institutional Review Board of the University of Tokushima. Written informed consent was obtained from all participants prior to blood sampling.

### Sequencing of the *KLF11* gene

To analyse the variants as previously reported and search for new ones, we screened all four exons, including exon/intron boundaries, 5′-untranslated region (UTR), 3′-UTR and 1000 bp upstream and downstream of *KLF11* with 24 randomly selected samples (12 control subjects and 12 cases). We partly used 96 additional case samples to confirm the variants.

The *KLF11* gene consists of four exons that span 11 kb of genomic DNA on chromosome 2p25. The total length of the re-sequencing region was about 8 kb. Coding regions and exon/intron boundaries were divided into 15 overlapping fragments and amplified by PCR. PCR primers were designed by Primer3 program (http://www-genome.wi.mit.edu/cgi-bin/primer/primer3_www.cgi) on the basis of the genomic contig sequence (gene ID: 8462, AC104794 and NM_003597). Details of the primers sequences are available on request.

PCR fragments were sequenced in both directions using the ABI BigDye Terminator chemistry method on an ABI 3100 or 3730xl automated sequencer (Applied Biosystems, Foster City, CA, USA). The sequencing conditions followed the protocol recommended by the manufacturer. The results were integrated using a SeqScape version 2.1.1 (Applied Biosystems), and individual single nucleotide polymorphisms (SNPs) were manually checked by two experienced researchers. Ambiguous base identifications were sequenced again, and SNPs were identified based on the sequence reported in GenBank.

### SNPs genotyping

Variants identified by either sequencing or databases were genotyped using the TaqMan Allelic Discrimination Assay (Applied BioSystems) on an ABI Prism 7700 (Applied BioSystems). Thermal cycling conditions followed the manufacturer's instructions. In our laboratory, genotyping showed 100% concordance with sequencing as previously described [13, 14].

### Statistical analysis

The statistical power was calculated using a PS power and sample-size program (http://biostat.mc.vanderbilt.edu/twiki/bin/view/Main/PowerSampleSize) and Genetic power calculator (http://pngu.mgh.harvard.edu/~purcell/gpc/). With a minor allele frequency (MAF) of 0.4, our sample size had 60–80% power to detect the SNPs with an odds ratio of 1.2–1.5 at a significance level of 0.05. With less frequent SNPs (assuming a MAF of 0.2), the same sample size showed lower power (57.4%) at the same significance level.

To test the association with Type 2 diabetes, four types of χ^2^-test (allele, genotype, dominant and recessive model) were calculated with SNPAlyze version 5.1 software (Dynacom, Yokohama, Japan). The crude odds ratio (OR) and 95% confidence interval (CI) were also calculated. To determine the significance level of the tests, nominal *P* values were corrected using Bonferroni's correction to avoid false-positive results. We corrected for 48 tests the total number of hypotheses (e.g. 11 SNPs multiplied by four models plus four haplotypes). We also used permutation testing as the correction for multiple testing (http://www.broad.mit.edu/mpg/haploview/). Distribution of a test statistic was estimated by analysing the statistics for a random sampling of 10 000 iterated permutations with a fixed total number of both cases and control subjects [15]. Deviations from Hardy–Weinberg equilibrium were examined using the χ^2^-test (SNPAlyze version 5.1), and results of *P* < 0.05 in control subjects were excluded from the analysis.

### Estimation of linkage disequilibrium (LD) and haplotype frequency

Simple pairwise LD coefficients, |D′| and *r*^2^, were calculated with SNPAlyze version 5.1 (Dynacom). This analysis was under the assumption of Hardy–Weinberg equilibrium. Haplotypes were inferred by the expectation–maximization (EM) algorithm. The differences of haplotype frequencies were calculated with χ^2^-tests and permutation tests. Distribution of a test statistic was estimated by analysing the statistics for a random sampling of 10 000 iterated permutations with a fixed total number of both cases and control subjects [15].

## Results

### Screening of the *KLF11* gene variants in Japanese populations

Based on the first report [6], we screened the genetic variants, including four major variants, Q62R in exon 2 (rs35927125), T220M in exon 3 (rs34336420), A347S in exon 3 (no dbSNP ID) and V395V in exon 3 (rs11687357). T220M and A347S are rare variants found only in MODY-like or early onset diabetes families. Q62R shows a MAF of 0.115 in their normoglycaemic group.

The results are shown in Table 2. With the re-sequencing effort, a total of eight variants were identified in 24 Japanese samples. These variants are believed to be common SNPs, showing a MAF of over 0.10 (0.104–0.354). Of eight variants, a novel A/C variant was detected in intron 3 without the register in the public database. Notably, there was no G allele of rs35927125 in the initial 24 samples and additional 96 case samples, although this mis-sense variant was strongly associated with Type 2 diabetes in the first report. In addition, we failed to detect two mis-sense variants (T220M and A347S) in 120 samples. There was no insertion or deletion. Within three zinc finger domains of the *KLF11* gene, we found no variants.

**Table 2:** Re-examination of the *KLF11* gene variants

					First 24 samples (12 controls and 12 cases)				Additional 96 samples (96 cases)			
						
Genome position	Variants previously reported (allele 1/2)	Location	dbSNP ID	Amino acid change	Allele 1	Hetero	Allele 2	Allele 2 frequency	Allele 1	Hetero	Allele 2	Allele 2 frequency
10 132 783	G/C	5′-flanking	No	No	24	0	0	0	95	0	0	0
10 132 891	Insertion (ACTTA)	5′-flanking	No	No	24	0	0	0	—	—	—	—
**10 133 094**	**G/A**	5′-flanking	rs4669520	No	18	5	1	0.146	65	29	2	0.172
10 133 162	Insertion (CT)	5′-flanking	No	No	24	0	0	0	96	0	0	0
10 133 418	Insertion (G)	5′-flanking	rs35035311	No	24	0	0	0	—	—	—	—
10 133 452	T/A	5′-flanking	No	No	24	0	0	0	—	—	—	−
10 134 357	Deletion (GCC)	5′-UTR	No	No	24	0	0	0	96	0	0	0
10 135 282	C/T	Intron 1	No	No	24	0	0	0	—	—	—	—
10 137 017	A/G	Exon 2	rs35927125	Q62R	24	0	0	0	96	0	0	0
10 138 131	G/C	Intron 2	No	No	24	0	0	0	—	—	—	—
10 138 721	C/T	Exon 3	rs34336420	T220M	24	0	0	0	96	0	0	0
10 139 101	G/T	Exon 3	No	A347S	24	0	0	0	96	0	0	0
**10 139 247**	**T/A**	Exon 3	rs11687357	V395V	18	5	1	0.146	65	27	2	0.165
**10 140 297**	**C/T**	Intron 3	rs6432052	No	18	5	1	0.146	67	27	2	0.161
**10 140 433**	**T/C**	Intron 3	rs6432053	No	11	9	4	0.354	53	35	8	0.266
**10 140 523**	A/C*	Intron 3	No	No	19	5	0	0.104	79	17	0	0.088
10 140 652	C/T	Intron 3	No	No	24	0	0	0	96	0	0	0
**10 140 713**	**G/A**	Intron 3	rs6721191	No	18	5	1	0.146	67	27	2	0.161
10 140 721	Deletion (A)	Intron 3	No	No	24	0	0	0	96	0	0	0
10 143 776	C/A	3′-UTR	rs4444493	No	24	0	0	0	—	—	—	—
**10 144 790**	**C/T**	3′-UTR	rs4669522	No	17	5	1	0.152	—	—	—	—
**10 145 085**	**C/T**	3′-UTR	rs7632	No	11	9	4	0.354	—	—	—	—

Genome position is based on the NCBI build 35. The eight variants identified in Japanese individuals, genome positions and variant types are shown in bold face.

* Novel A/C variant in intron 3 was detected in this study.

*KLF11*, Krüppel-like factor 11; NCBI, National Left for Biotechnology Information; UTR, untranslated region.

### Association study with the genetic variants in *KLF11*

As a result of screening, nine TaqMan probes were designed within the coding region of *KLF11*, in which Q62R (rs35927125) was included to examine its genotypes in more samples. To estimate LD around the *KLF11* gene, we placed three additional TaqMan probes (rs4073397, rs3885668 and rs10171924) outside the coding region with the HapMap JPT database as a reference (http://www.hapmap.org). These SNPs covered the region of interest with, on average, a less than 10-kb interval. All together, 12 TaqMan probes were designed for genotyping. After removing rs6432052, which failed to be successfully genotyped using the TaqMan method, the remaining 11 variants, including rs35927125, were genotyped in 1818 samples. The concordance rate of duplicate genotypes was over 99%. The genotyping success rate was between 98.5–100%.

The results of genotyping and association with Type 2 diabetes are shown in Table 3. The genotype distribution was in the Hardy–Weinberg equilibrium (*P* > 0.05). In all 1818 samples, there was no G allele in rs35927125. No SNPs across the coding region of the *KLF11* gene showed a significant association in the initial 731 samples (from the Tokushima district). This result was replicated in larger cohorts with 524 control subjects (from PSC) and 563 Type 2 diabetic patients (from the Kansai district). In a joint analysis of all samples, no significant association of diabetes was observed with any SNPs, including a novel A/C variant in intron 3. This SNP was not studied in either the first [6] or the second report of Caucasian [10]. In two Japanese controls, there was no significant difference in allele frequencies, suggesting no harmful biases in the two control groups (Supplementary Table 1).

**Table 3:** Genotype and allele frequencies for 11 SNPs in 1818 Japanese individuals

						Genotype frequency				Minor allele frequency						Hardy-Weinberg equilibrium test
													
Genome position	Variants Allele 1/2	Location	dbSNP ID	Information of HapMap JPT	Amino acid change	Genotype	Control (*n* = 893)	Case (*n* = 925)	*P* value*	Control (*n* = 893)	Case (*n* = 925)	*P* value**	Permutation *P* value	Odds ratio	95% CI	*P* value
10 112 635	G/A	5′-flanking	rs4073397	Available	No	GG	259 (0.29)	216 (0.23)	0.021	0.462	0.504	0.010	0.046	1.186	1.04–1.35	0.90
						GA	439 (0.49)	479 (0.52)								
						AA	191 (0.21)	224 (0.24)								
10 129 077	T/C	5′-flanking	rs3885668	Available	No	TT	636 (0.71)	640 (0.70)	0.468	0.156	0.170	0.283	0.710	1.101	0.93–1.32	0.86
						TC	233 (0.26)	248 (0.27)								
						CC	23 (0.03)	32 (0.03)								
10 133 094	G/A	5′-flanking	rs4669520	No	No	GG	636 (0.71)	636 (0.69)	0.571	0.157	0.170	0.291	0.719	1.100	0.92–1.32	0.70
						GA	232 (0.26)	252 (0.27)								
						AA	24 (0.03)	30 (0.03)								
10 137 017	A/G	Exon 2 +143	rs35927125	No	Q62R	AA	893 (1.00)	925 (1.00)	—	—	—	—	−—	—	—	—
						AG	0	0								
						GG	0	0								
10 139 247	T/A	Exon 3 +873	rs11687357	No	V395V	TT	635 (0.72)	633 (0.70)	0.518	0.152	0.166	0.272	0.687	1.106	0.92–1.33	0.77
						TA	224 (0.25)	241 (0.27)								
						AA	22 (0.02)	29 (0.03)								
10 140 433	T/C	Intron 3 +1113	rs6432053	Available	No	TT	442 (0.50)	457 (0.50)	0.880	0.292	0.296	0.758	0.998	1.023	0.89–1.18	0.78
						TC	370 (0.42)	381 (0.41)								
						CC	73 (0.08)	82 (0.09)								
10 140 523	A/C	Intron 3 +1203	no	No	No	AA	749 (0.84)	765 (0.83)	0.331	0.082	0.091	0.331	0.775	1.122	0.89–1.41	0.83
						AC	136 (0.15)	146 (0.16)								
						CC	5 (0.01)	11 (0.01)								
10 140 713	G/A	Intron 3 +1393	rs6721191	No	No	GG	638 (0.72)	643 (0.70)	0.522	0.154	0.167	0.269	0.680	1.105	0.93–1.32	0.89
						GA	229 (0.26)	250 (0.27)								
						AA	22 (0.02)	29 (0.03)								
10 144 790	C/T	3′-UTR +1558	rs4669522	Available	No	CC	640 (0.72)	643 (0.70)	0.463	0.154	0.168	0.241	0.637	1.112	0.93–1.33	0.93
						CT	231 (0.26)	251 (0.27)								
						TT	22 (0.02)	30 (0.03)								
10 145 085	C/T	3′-UTR +1853	rs7632	Available	No	CC	443 (0.50)	454 (0.50)	0.768	0.290	0.296	0.671	0.990	1.032	0.89–1.19	0.78
						CT	368 (0.42)	374 (0.41)								
						TT	72 (0.08)	83 (0.09)								
10 152 112	G/A	3′-flanking	rs10171924	Available	No	GG	634 (0.71)	640 (0.70)	0.483	0.156	0.166	0.399	0.850	1.080	0.90–1.29	0.99
						GA	235 (0.26)	243 (0.27)								
						AA	21 (0.02)	30 (0.03)								

Genome position is based on the NCBI build 35.

*P* values for

* genotype frequency

** allele frequency. Permutation *P* value are calculated for allele frequency.

Hardy–Weinberg equilibrium is calculated using only control subjects.

The total of genotype frequencies does not reach 1.0 because undetermined results were excluded from this table.

CI, confidence interval; NCBI, National Left for Biotechnology Information; SNP, single nucleotide polymorphism.

With rs4073397 located 21 kb upstream from a start codon, the nominal *P* value showed a weak association (*P* = 0.010, allele model). However, this value was not significant after adjustment with Bonferroni's correction (corrected for 48 tests), although a marginal association was observed with permutation testing (permutation *P* = 0.046). When the analysis was separated by initial and replication tests, neither showed significant results (*P* = 0.086 and 0.058, allele model) (Supplementary Tables 2 and 3).

In this study, case–control samples were not completely matched for age. To examine whether this bias affected the analysis, 446 control samples under the age of 35 years were excluded. The genotyping data were reanalysed with 447 control subjects over the age of 35 years and 925 cases. Once again, none of the SNPs was significant (*P* > 0.05), but rs4073397 showed nominal association with Type 2 diabetes (*P* = 0.004, allele model). This value was not significant after adjustment for multiple testing, but it should be noted that this sample size had considerably reduced power (41.6%).

As a result, statistical significance, assessed using a correction for multiple testing, showed no association with Type 2 diabetes, except for rs4073397 with a marginal association.

### LD estimation and haplotype-based association study

**FIGURE 1:**
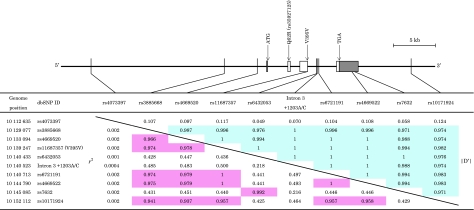
Genomic structure of *KLF11* and pairwise LD values with 10 verified SNPs across the *KLF11* gene. In the upper part, the *KLF11* gene structure and the positions of 10 SNPs are indicated based on the NCBI human build 35. Exons are shown as boxes. Coding sequences are represented as white boxes, and untranslated regions as grey boxes. Introns and flanking sequences are shown as lines connecting the boxes. Two major variants (Q62R and V395V), start (ATG) and stop (TGA) codons are indicated as arrows. In the lower part, simple pairwise LD values, |D′| (right) and *r*^2^ (left), were calculated with the SNPAlyze version 5.1 (Dynacom) using 893 control samples. Blue and red columns indicate the values over 0.9 with |D′| and *r*^2^. *KLF11*, Krüppel-like factor 11; LD, linkage disequilibrium; NCBI, National Center for Biotechnology Information; SNP, single nucleotide polymorphism.

As shown in Fig. 1, simple pairwise LD values, quantified by |D′| and *r*^2^, were calculated in 893 control subjects. With the definition of |D′|, single strong LD values (|D′| > 0.9) were found within nine SNPs (from rs3885668 to rs10171924) across the *KLF11* gene. In contrast, two haplotype blocks were found: one including rs3885668, rs4669520 and rs11687357 and the other rs6721191 and rs4669522, with **r*^2^* values > 0.9. The total frequency of all haplotypes was over 98%.

In the analysis of |D′|, we found no haplotype-based association with Type 2 diabetes in either the initial (Tokushima district) or replication samples (Kansai district). In the joint analysis of all samples, the result was also non-significant (Table 4). Additionally, there was no significant association of different haplotypes with the definition of *r*^2^ (data not shown).

**Table 4:** Haplotype analyses with nine SNPs defined by |D′|

		Number of haplotypes and frequencies				
				
Haplotypes	SNPs 123456789	Control (frequency)	Case (frequency)	Overall (frequency)	*P* value	Permutation *P* value
1	TGTTAGCCG	1256 (0.703)	1294 (0.699)	2550 (0.701)	0.803	0.825
2	TGTCAGCTG	238 (0.133)	230 (0.124)	468 (0.129)	0.421	0.430
3	CAACCATTA	143 (0.080)	168 (0.091)	311 (0.086)	0.247	0.265
4	CAACAATTA	126 (0.071)	132 (0.071)	258 (0.071)	0.925	0.951
Total		(> 0.99)	(> 0.99)	(> 0.99)		

SNP 1 is equivalent to rs3885668 (T/C), SNP 2 to rs4669520 (G/A), SNP 3 to rs11687357 (T/A), SNP 4 to rs6432053 (T/C), SNP 5 to intron 3 +1203 (A/C), SNP 6 to rs6721191 (G/A), SNP 7 to rs4669522 (C/T), SNP 8 to rs7632 (C/T) and SNP 9 to rs10171924 (G/A).

The total frequencies of haplotypes do not reach 1.0 because rare haplotypes with frequencies under 0.05 are excluded.

*P* values are calculated by χ^2^-tests between case and control subjects with the permutation method.

SNP, single nucleotide polymorphism.

## Discussion

As the *KLF11* gene is one of the key regulators in insulin secretion, we considered *KLF11* to be an important candidate gene for Type 2 diabetes and systematically surveyed the genetic variants. Although common alleles with an OR of 1.2–1.5 could be detected with 60–80% power with prior simulation, we could not find any strong evidence with single–SNPs association tests and haplotype analyses in 1818 Japanese subjects. However, the effect of less frequent SNPs might be underestimated, as our power to detect less frequent SNPs was much less. It is therefore important to conduct a meta-analysis and/or larger clinical trial to detect the modest effect of less frequent variants in this gene [16, 17].

When identifying the disease-susceptibility variants, type 1 error (false positive) is of particular importance, and needs to be minimized by statistical methods [18]. We used Bonferroni's correction to adjust the significance level in disease association. We also used the permutation method to control for a type 1 error. Even after these two analyses, our data do not provide strong evidence that the *KLF11* variant is associated with Type 2 diabetes in our Japanese population.

Nevertheless, there might be a marginal association with rs4073397 located 21 kb upstream from the coding region and outside the LD block defined by |D′|. With the hepatocyte nuclear factor 4α (*HNF4α*) gene, the pancreas-specific transcription start site is located 45 kb upstream relative to that of the liver form, and SNPs within the distal promoter region showed significant association with Type 2 diabetes [19, 20]. We could not completely exclude the possibility of weak genetic contributions in the putative regulatory region of the *KLF11* gene, but the actual mechanism of how the variant would affect the regulation of *KLF11* remains to be elucidated.

Although the contribution of genetic factors to the risk of Type 2 diabetes was extensively examined, it remains unclear how the discrepancy arose in studies that analysed the disease-susceptibility variants [21]. In the first report, the G allele in rs35927125 showed the significant association, but this risk allele was completely absent in Japanese individuals. In addition, a recent large study failed to find an association of the Q62R-allele in an independent Caucasian cohort [10]. In previous association studies [22–24], a similar case showed that the frequency of K121Q variant in ectoenzyme nucleotide pyrophosphate phosphodiesterase 1 (*ENPP1*) was quite different among the ethnic groups. It is possible that the disease-susceptibility induced by variants is modulated by complicated parameters, such as other ethnic specific genetic and/or environmental factors [25].

In conclusion, we were unable to find any association of common *KLF11* variants with Type 2 diabetes in 1818 Japanese subjects, although a larger study may be required to completely exclude the potential effect of rare variants on this gene. The present association study contributes to the future conclusive results of *KLF11* variants in different populations.

## Abbreviations

CI: confidence interval
*KLF11*: Krüppel-like factor 11
LD: linkage disequilibrium
MAF: minor allele frequency
MODY: maturity-onset diabetes of the young
OR: odds ratio
SNP: single nucleotide polymorphism
UTR: untranslated region

## Supplementary material

This material is available as part of the online article from: http://www.blackwell-synergy.com/doi/abs/10.1111/j.1464-5491.2007.02315.x

(This link will take you to the article abstract).
